# Vitamin D_3_ Activates Phosphatidylinositol-3-Kinase/Protein Kinase B via Insulin-Like Growth Factor-1 to Improve Testicular Function in Diabetic Rats

**DOI:** 10.1155/2019/7894950

**Published:** 2019-06-04

**Authors:** Yanyan He, Yang Liu, Qing-Zhu Wang, Feng Guo, Fengjuan Huang, Linlin Ji, Tingting An, Guijun Qin

**Affiliations:** ^1^Division of Endocrinology, Department of Internal Medicine, The First Affiliated Hospital of Zhengzhou University, Zhengzhou 450052, China; ^2^Institute of Clinical Medicine, The First Affiliated Hospital of Zhengzhou University, Zhengzhou 450052, China

## Abstract

**Objective:**

In diabetes mellitus, vitamin D_3_ deficiency affects sex hormone levels and male fertility; however, the mechanism leading to the disorder is unclear. This research was designed to investigate the mechanism of vitamin D_3_ deficiency and hypogonadism in diabetic rats. Our aim was to assess serum vitamin D_3_ levels and the relationship among vitamin D_3_, insulin-like growth factor-1 (IGF-1), and testicular function.

**Materials and Methods:**

Rats with streptozotocin-induced diabetes were randomly divided into four groups and treated with different doses of vitamin D_3_: no vitamin D_3_, low (0.025 *μ*g/kg/day), high (0.1 *μ*g/kg/day), and high (0.1 *μ*g/kg/day) with JB-1 (the insulin-like growth factor-1 receptor inhibitor group, 100 *μ*g/kg/day). The groups were compared with wild-type rats, which function as the control group. Various parameters such as vitamin D_3_ and IGF-1 were compared between the experimental and wild-type groups, and their correlations were determined.

**Results:**

Twelve weeks of vitamin D_3_ supplementation improved the testosterone levels, as shown by the increase in the level of serum IGF-1 in diabetic rats. Phosphatidylinositol-3-kinase (PI3K)/protein kinase B (AKT), which was a downstream of the signaling pathway of IGF-1, was significantly increased after vitamin D_3_ treatment.

**Conclusions:**

The study shows that vitamin D_3_ may promote the expression of testosterone and improve testicular function in diabetic rats by activating PI3K/AKT via IGF-1.

## 1. Introduction

Diabetes mellitus is a metabolic and endocrine disorder characterized by hyperglycemia and glucose intolerance [1]. Long-term uncontrolled diabetes mellitus with sustained high blood glucose levels causes hypogonadism, testicular damage, sexual dysfunction, and erectile dysfunction [2–6]. Studies have shown that hypogonadism is significantly more frequent in male patients with diabetes than in those without diabetes, and in male patients with diabetes, free testosterone and total testosterone levels are reduced by 46% and 34%, respectively [7].

In male rodents, hyperglycemia affects testicular Leydig cells and pituitary gonadotropin cells, resulting in the decreased synthesis of testosterone (T), luteinizing hormone (LH), and follicle-stimulating hormone (FSH) levels, leading to reduced sperm motility and vitality [8, 9]. Oxidative stress, advanced glycation end products (AGEs), abnormal cell metabolism, and other disorders are major causes of diabetic hypogonadism resulting from long-term hyperglycemia [10].

1*α*,25-Dihydroxy vitamin D_3_ (1,25-[OH]_2_D_3_), the active form of vitamin D_3_, binds to the vitamin D receptor (VDR) to regulate calcium and phosphorus metabolism [11]. Besides, researches have found that vitamin D_3_ can affect the progress of many diseases through multiple pathways [12–14]. Vitamin D_3_ might have an impact on the hypogonadism of DM [13, 15] with increasing evidence suggesting that VDR is distributed in the testes, vas deferens, and spermatozoa [15]. Studies show that vitamin D_3_ deficiency can affect sperm quality and quantity and sex hormone levels and can lead to male infertility [16, 17]. Studies also have confirmed that insulin-like growth factor-1 (IGF-1) can stimulate testosterone production by testicular interstitial cells [18], and vitamin D_3_ supplementation can increase the levels of IGF-1 in the circulation [19].

Whether vitamin D_3_, insulin-like growth factor-1, and hypogonadism in diabetic rats are related is unclear. In this study, the mechanism of testicular function in diabetic rats and the effects of vitamin D_3_ treatment were evaluated. Therefore, we speculated that IGF-1 affects testicular function via the phosphatidylinositol-3-kinase (PI3K)/protein kinase B (AKT) signaling pathway, a downstream signaling pathway of IGF-1 in diabetic rats [20]. We studied the effects of vitamin D_3_ supplementation on the testis of diabetic rats and explored the pathway to regulate its biological effects in order to improve the level of clinical treatment.

## 2. Materials and Methods

### 2.1. Animals and Treatment

Specific pathogen-free, 8-week-old male Sprague–Dawley rats weighing approximately 200 g (Experimental Animal Center of Henan Province, Zhengzhou, China) were housed in individual ventilated cages, with free access to food and water. All experimental procedures were conducted in accordance with the First Affiliated Hospital of Zhengzhou University guidelines for the care and use of laboratory animals and were approved by the University Animal Care and Use Committee. After 12 h of fasting, rats were injected once intraperitoneally with a solution of 60 mg/kg streptozotocin (STZ, Sigma-Aldrich, St. Louis, MO, USA) in fresh 0.05 M citrate buffer (pH 4.5) to induce diabetes. Six rats that received an injection of diluent buffer alone served as the normal control group (CG group). Blood glucose (BG) was measured using a blood glucose meter (Roche Company, Basel, Switzerland), and animals with fasting blood glucose higher than 16.7 mmol/L were considered diabetic. Diabetic rats were randomly divided into four groups and treated with different doses of vitamin D_3_: no vitamin D_3_ (DM), rats treated with a low dose of active vitamin D_3_ (LD; 0.03 *μ*g/kg/d), rats treated with a high dose of active vitamin D_3_ (HD; 0.1 *μ*g/kg/d), and rats treated with a high dose of vitamin D_3_ and the insulin-like growth factor-1 receptor (IGF-1R) inhibitor (inhibitor group; JB-1 100 *μ*g/kg/d), with 6 rats per group. Each vitamin D_3_ group received a dose of 1,25-(OH)_2_D_3_ which was determined by consulting the literature and from previous experiments [21, 22]. All groups were treated with 0.1 mL/d peanut oil by oral gavage, and the inhibitor group was treated with JB-1 at 100 *μ*g/kg/d subcutaneously [23, 24].

### 2.2. Measurement of Body Weight and Testis Weight and the Collection of Serum and Testicular Tissues

After 12 weeks, body weight (BW), testicular weight (TW), and blood glucose levels were determined. After anesthesia with 10% chloral hydrate, cardiac blood was collected and centrifuged at 4°C for 2 min at 3000 rpm. Serum was stored at -80°C. The testes were weighed after surgical resection. Few of the testes were stored in liquid nitrogen for further analysis. The remaining were fixed for histological assessments.

### 2.3. Determination of IGF-1, T, 1,25-(OH)_2_D_3_, Calcium (Ca), and Phosphorus (P)

Serum IGF-1, 1,25-(OH)_2_D_3_, and T levels were detected using an enzyme-linked immunosorbent assay (ELISA) kit (Shanghai AMEKO Biological Technology, Shanghai, China), following the kit instructions and previously described methods [25]. Ca and P levels in the serum were analyzed using the Roche Cobas 8000 Automatic Biochemical Analyzer and an ELISA kit (Nanjing Jiancheng Bioengineering Institute, Nanjing, China).

### 2.4. Light and Transmission Electron Microscopy

The sections were embedded in paraffin and stained with hematoxylin-eosin. The pathology of testicular tissues was examined by light microscopy. Testes were fixed with 1% osmic acid and dehydrated with ethyl alcohol and acetone. Then, the samples were placed in epoxy resin liquid for embedding, sectioning, and staining with uranium acetate. The tissues were observed under a transmission electron microscope (H-7500; HITACHI, Tokyo, Japan). Immunohistochemical staining was performed according to previously described methods [26]. Antibodies at the following dilutions: anti-VDR (1 : 2000) and anti-IGF-1R (1 : 30), were used. Protein expression was quantified using ImageJ (NIH, Bethesda, MD, USA).

### 2.5. Ribonucleic Acid Extraction and Quantitative Reverse Transcription Polymerase Chain Reaction (qRT-PCR)

The *IGF-1R* and *VDR* mRNA expression levels in the rat testes were detected by qRT-PCR. Total ribonucleic acid (RNA) was extracted from tissues by TRIzol (TaKaRa Bio, Shiga, Japan), and complementary deoxyribonucleic acid (cDNA) was synthesized according to the manufacturer's instructions (GeneCopoeia, Rockville, MD, USA). The expression of the target genes was performed using KOD SYBR Green qPCR Mix Kit (GeneCopoeia) with appropriate primers on the Applied Biosystems 7500 FAST system (Foster City, CA, USA). The primers were obtained from GeneCopoeia. The expression levels of the genes were measured relative to the *GAPDH* levels and were evaluated using the 2^-*ΔΔ*CT^ method. Primer sequences are presented in Table 1.

### 2.6. Western Blot Analysis

The levels of VDR, IGF-1R, PI3k, AKT, phosphorylated-PI3K (p-PI3K), and phosphorylated-AKT (p-AKT) in the testes were detected by western blot technique following previously described methods by our group and others [27, 28]. The VDR antibody was obtained from Abcam (Cambridge, UK). Antibodies against PI3K, AKT, p-PI3K, and p-AKT were purchased from CST (Danvers, MA, USA). Western blotting was performed according to standard methods using antibodies at the following dilutions: anti-VDR, 1 : 500; anti-PI3K, 1 : 1000; anti-*β*-actin, 1 : 1000; anti-IGF-1R, 1 : 1000; anti-AKT, 1 : 1000; anti-p-PI3K, 1 : 1000; and anti-p-AKT, 1 : 1000. Immunohistochemical signals were quantified using ImageJ (NIH, Bethesda, MD, USA).

### 2.7. Statistical Analysis

Results were analyzed by SPSS 21.0 (IBM, Endicott, NY, USA). All the results were presented as means ± S.D. Comparisons among more than two groups were evaluated by one-way ANOVA, followed by Bonferroni tests. *p* < 0.05 was considered statistically significant.

## 3. Results

### 3.1. General Condition of Animals and Biochemical Analyses

At the end of 12 weeks, the BW was approximately 40.7% lower in the DM group than in the CG group and the BG levels in the DM group were significantly increased as compared to those in the beginning of the experiment (*p* < 0.05). Test statistics and *p* values indicate significant differences. There were significant differences between the DM and CG groups in TW (Table 2). There was no significant difference in serum Ca or P levels between the CG and DM groups (*p* > 0.05). After vitamin D_3_ treatment, the T levels in the HD and LD groups were significantly greater than those in the DM group (*p* < 0.05) and there was no significant difference between the HD and LD groups (*p* > 0.05). Additionally, IGF-1 and vitamin D_3_ levels were significantly higher in the LD group than in the DM group (*p* < 0.05; Table 2).

### 3.2. Pathological Changes in the Testis

**Figure 1:**
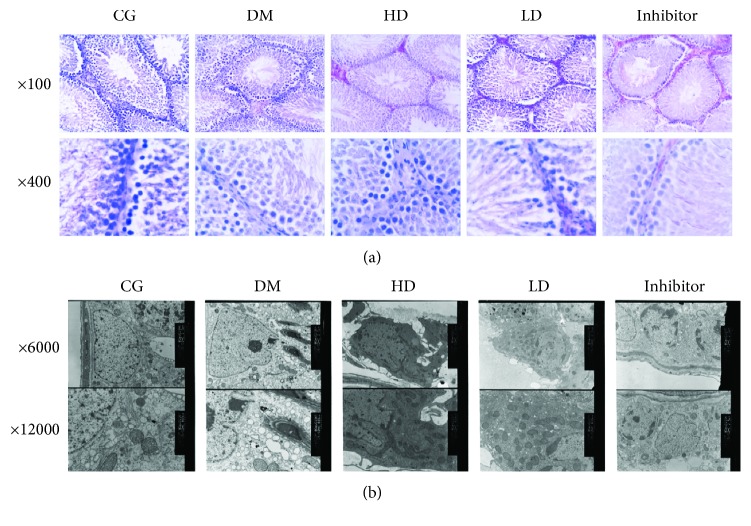
(a) Histopathological changes in testicular tissue under a light microscope (stained with hematoxylin and eosin dye, ×100, ×400). (b) Histopathological changes in testicular tissue under a transmission electron microscope (×6000, ×12000). Histological assessment under a light microscope showed that testicular tissues from the DM group exhibited extensive, loose interstitial cells and visible interstitial atrophy. The testicular tissue sections of the HD and LD groups exhibited slightly more active, spermatogenic cells that were arranged more closely, and a more significant number of interstitial cells. The histological properties of the CG group were superior to those of the above three groups. Based on transmission electron microscopy, the organelles of the testis tissue exhibited atrophy; the mitochondria, endoplasmic reticulum, and other organelles exhibited vacuoles; and the mitochondrial ridge was widened and dissolved. These changes were not observed in the interstitial cells in the CG group. The histological properties in the HD and LD groups showed altered expressions levels between the CG and DM groups. The level measured in the inhibitor group was slightly lower than that in the HD group.

Based on histological assessment under the light microscope, the testicular tissues in the DM group exhibited extensive, loose interstitial, and obvious interstitial atrophy. After vitamin D_3_ treatment, the testicular tissue sections of the HD and LD groups were slightly more active, the spermatogenic cells were arranged more closely, and there were more interstitial cells. The histological properties of the CG group were better than those of the above three groups (Figure 1(a)). By transmission electron microscopy, the organelles of the testis tissue exhibited atrophy; the mitochondria, endoplasmic reticulum, and other organelles exhibited vacuoles; and the mitochondrial ridge became wider and dissolved. These changes were not observed in the interstitial cells in the CG group. The histological properties in the HD and LD groups showed differential expression between the CG and DM groups. The testicular tissues of the inhibitor group were slightly lower than that of the HD group (Figure 1(b)).

### 3.3. Determination of VDR

**Figure 2:**
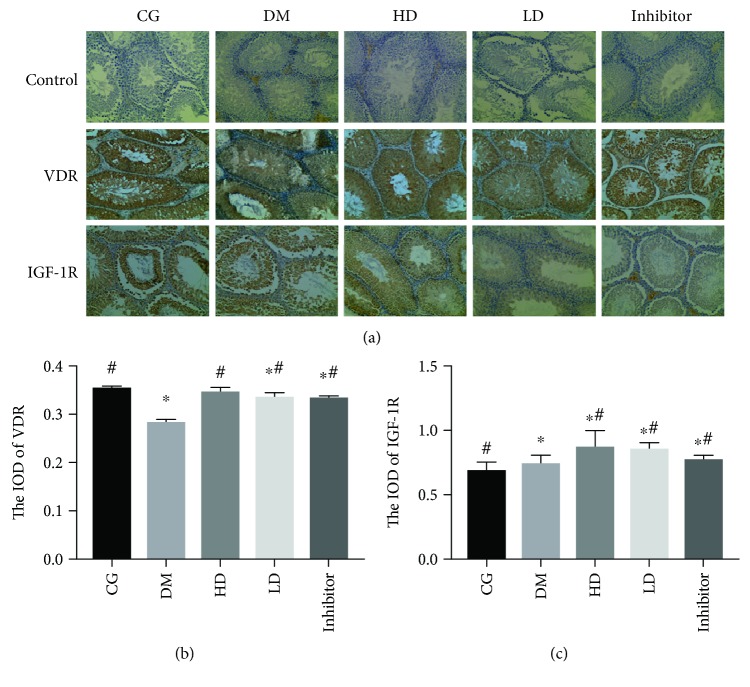
(a) Immunohistochemical analysis of the expression of VDR and IGF-1R in each group (×100). (b, c) Immunohistochemical analyses of VDR and IGF-1R in each group. IOD: integrated optical density. In groups treated with vitamin D, VDR expression was more strongly positive than that in the DM group. Compared with the DM group, the integrated optical density for other groups was significantly different (*p* < 0.05). There were no significant differences in VDR expression among the LD, HD, and CG groups (*p* > 0.05). Immunohistochemical analysis showed that the expression of IGF-1R in the DM group was significantly lower than that in the CG, HD, and LD groups (*p* < 0.05). ^∗^*p* < 0.05 versus the CG group; ^#^*p* < 0.05 versus the DM group.

**Figure 3:**
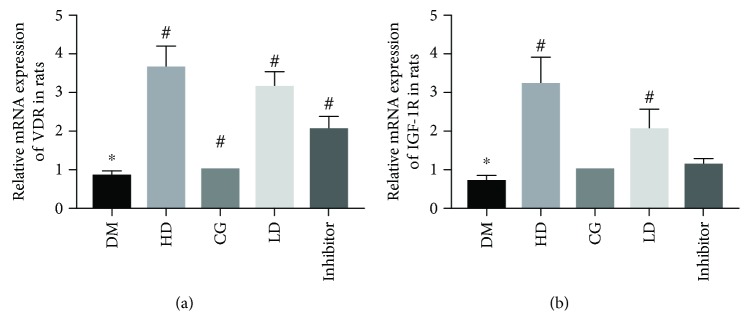
Expression of *VDR* and *IGF-1R* mRNA in rat testicles in each group. The RT-PCR results showed that the mRNA levels of *VDR* and *IGF-1R* in the DM group were significantly lower than those in the CG group (*p* < 0.05). The expression levels of *VDR* and *IGF-1R* in the HD and LD groups were significantly higher than that in the DM group (*p* < 0.05). The expression of *IGF-1R* in the LD group was significantly higher than that in the DM group (*p* < 0.05). The expression of *IGF-1R* in the inhibitor group was significantly lower than that in the HD group. ^∗^Compared with the CG group, *p* < 0.05; ^#^compared with the DM group, *p* < 0.05.

**Figure 4:**
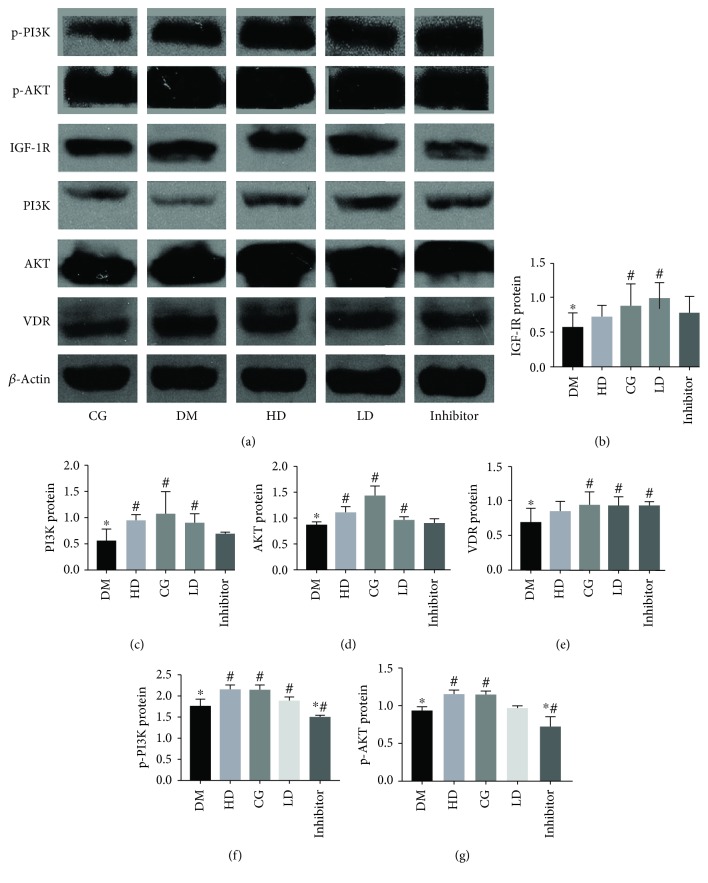
Protein expression levels of VDR, IGF-1R, PI3K, AKT, p-PI3K, and p-AKT in the rat testis. The protein expression levels of VDR and IGF-1R in the DM group were significantly lower than those in the CG group (*p* < 0.05). The expression levels of VDR in the HD and LD groups were significantly higher than that in the DM group (*p* < 0.05). The expression of IGF-1R in the LD group was significantly higher than that in the DM group (*p* < 0.05). The expression of IGF-1R in the inhibitor group was significantly lower than that in the HD group. The expression levels of PI3K, AKT, p-PI3K, and p-AKT differed significantly between the DM and CG groups (*p* < 0.05). The expression levels of PI3K, AKT, p-PI3K, and p-AKT in the group treated with vitamin D were significantly higher than those in the DM group (*p* < 0.05). The expression levels of PI3K, AKT, p-PI3K, and p-AKT were significantly different between the HD group and the inhibitor group (*p* < 0.05). ^∗^Compared with the CG group, *p* < 0.05; ^#^compared with the DM group, *p* < 0.05.

Using immunohistochemical analyses, VDR-positive cells were detected by brown or tan staining by microscope. VDR expression was more strongly positive in groups treated with vitamin D_3_ than in the DM group. Compared with the DM group, the integrated optical density of the other groups was significantly different (*p* < 0.05). There was no significant difference in the VDR expression among the LD, HD, and CG groups (*p* > 0.05; Figure 2). The qRT-PCR and western blotting results showed that the levels of *VDR* in the DM group were significantly lower than those in the CG group (*p* < 0.05). The expression levels of *VDR* in the HD and LD groups were significantly higher than that in the DM group (*p* < 0.05; Figures 2–4).

### 3.4. Vitamin D_3_ Promotes IGF-1 and PI3K/AKT Expression in Diabetic Rats

Immunohistochemical analysis showed that the expression of IGF-1R in the DM group was significantly lower than that in the CG, HD, and LD groups (*p* < 0.05). The mRNA level of *IGF-1R* in the DM group was significantly lower than those in the CG group (*p* < 0.05). The expression of *IGF-1R* in the LD group was significantly higher than that in the DM group (*p* < 0.05). The expression levels of PI3K, AKT, p-PI3K, and p-AKT differed significantly between the DM and CG groups (*p* < 0.05). The expression levels of PI3K, AKT, p-PI3K, and p-AKT in the group treated with vitamin D_3_ were significantly higher than those in the DM group (*p* < 0.05). The expression of IGF-1R in the inhibitor group was significantly lower than that in the HD group. The expression levels of PI3K, AKT, p-PI3K, and p-AKT were significantly different between the HD and inhibitor groups (*p* < 0.05; Figures 2–4).

## 4. Discussion

The results of the present study demonstrated that persistent hyperglycemia could lead to a decrease in spermatogenic vitality and testosterone levels in diabetic rats. Active vitamin D_3_ exerted a protective effect on testicular function against damage induced by diabetes, including protection against changes in spermatogenesis and testosterone biosynthesis. We found that vitamin D_3_ promotes gonad function through activating PI3K/AKT via IGF-1 signaling.

Similar mechanisms underlying hypogonadism in DM included oxidative stress, inflammatory responses, increased apoptosis, androgen levels, microvascular disease, and neuropathy [29]. In the STZ-induced DM animal models, changes in testicular histology, alterations in the number and activity of spermatozoa, and decreased testosterone levels were observed, which were consistent with previous results [22, 30]. Previous studies showed that the pathogenesis of gonadal dysfunction caused by diabetes may be related to histological changes of testis [31]. T synthesis mainly depended on interstitial cells. Consistent with previous studies, we observed morphological changes in Leydig cells, accompanied by changes in testosterone levels, which were important causes of hypogonadism in male diabetic rats [29].

We found that vitamin D_3_ can improve testicular function in diabetic rats. How does vitamin D_3_ play a protective role in diabetic rats? The effect of serum vitamin D_3_ on reproductive function depends on the presence of VDR in the testicular tissues, genital prostate tract, and spermatozoa [15]. Dihydroxyvitamin D_3_ is a hormone that regulates several essential physiological and biochemical functions, such as calcium and phosphorous homeostasis as well as cellular growth, differentiation, and apoptosis. Vitamin D_3_ interacts with the selective VDR ligand binding pocket (VDR-gp) in the cell membrane or cytoplasm of the reproductive system. VDR forms a complex structure with retinoid X receptor (RXR) and binds to the vitamin D_3_ response element (VDRE) within the target gene promoter region, thereby regulating the transcription of genes involved in cell proliferation and differentiation [32]. In addition to its classical role as a transcription regulator, VDR has been shown to exert a nongenomic effect in the male reproductive system via second messengers, ion channels, or protein phosphorylation. Other animal experiments have confirmed that vitamin D deficiency in male animals may result in decreases in mating ability, spermatogenic capacity, and T levels. In this study, the level of T improved after supplementation with vitamin D_3_. Also, we showed that in the STZ-induced DM animal model, the expression of VDR in the DM group was lower than the control group. Similar results were found in previous studies [33, 34]. This result may be due to oxidative stress, inflammation, increased apoptosis, hyperglycemia, and other factors leading to the downregulation of the expression of VDR. Additionally, Khan et al. showed that the low-level expression of VDR in the DM group might be related to vitamin D_3_ deficiency [35].

We also observed that plasma IGF-1 levels were elevated in diabetic rats after oral vitamin D_3_ supplementation. IGF-1 and its receptor provide a potent proliferative signaling system that stimulates growth in many cell types and blocks apoptosis. Recent studies reported that serum IGF-1 binds to IGF-1R on Leydig cells and promotes the synthesis of testosterone by Leydig cells via a series of reactions [30], and the addition of IGF-1 can improve sperm motility [36, 37]. Previous experiments have shown that there are fewer testicular Leydig cells in serum IGF-1-deficient mice than in normal mice [38] and exogenous supplementation with IGF-1 could promote Leydig cell proliferation and differentiation [39]. Exogenous vitamin D_3_ supplementation increases the blood levels of IGF-1. Vitamin D_3_ is highly likely to regulate IGF-1 concentrations by acting in the liver because this organ is the primary source of circulating IGF-1 [40]. However, its specific mechanism of action is not yet clear. In this study, vitamin D_3_ increased the level of serum IGF-1 in DM rats and PI3K/AKT was activated. The application of the IGF-1R inhibitor JB-1 blocked the PI3K/AKT signaling pathway and reduced spermatogenesis and testosterone synthesis in the testes. Therefore, IGF-1 can regulate spermatogenesis and testicular function.

In conclusion, vitamin D_3_ could increase the level of IGF-1 in diabetic rats and improve gonadal function via the PI3K/AKT pathway. However, the optimal dose of vitamin D_3_ for diabetic rats is unclear. In this study, the gradient of vitamin D_3_ doses was small, and future studies should examine the relationship between IGF-1 and the dose of vitamin D_3_.

## Figures and Tables

**Table 1:** Primer sequences for qRT-PCR.

Gene	Primer sequences
*VDR*	Forward: 5′-CACAGGCTTCCACTTCAATGCTA-3′ Reverse: 5′-TCATGCCGATGTCCACACAG-3′

*IGF-1R*	Forward: 5′-CTCGGCATCAAACTCCTC-3′ Reverse: 5′-CTTTATCACCACCGCACA-3′

*GAPDH*	Forward: 5′-CATTCTTCCACCTTTGAT-3′ Reverse: 5′-CTGTAGCCATATTCATTGT-3′

**Table 2:** Blood glucose (BG), body weight (BW), testicular weight (TW), and serum biochemical analysis at week 12.

Group	BG (mmol/L)	BW (g)	TW (g)	Ca (mmol/L)	P (mmol/L)	T (mmol/L)	IGF-1 (ng/mL)	Vitamin D_3_ (*μ*g/mL)
DM	29.85 ± 2.39^∗^	276.5 ± 85.22^∗^	1.48 ± 0.11^∗^	2.33 ± 0.03	3.02 ± 1.13	559.26 ± 59.15^∗^	5.45 ± 1.40^∗^	7604.13 ± 273.65^∗^
HD	27.95 ± 2.92^∗^	278.00 ± 30.00^∗^	1.53 ± 0.15^∗^	2.44 ± 0.09	2.71 ± 0.27	629.46 ± 17.94^#^	6.12 ± 2.06^∗^	8293.63 ± 450.93^#^
LD	27.13 ± 3.10^∗^	284.60 ± 41.24^∗^	1.49 ± 0.18^∗^	2.36 ± 0.04	2.18 ± 0.20	619.76 ± 40.81^#^	12.7 ± 4.94^∗^^#^	8043.32 ± 192.10^#^
Inhibitor	28.50 ± 5.17^∗^	275.94 ± 20.10^∗^	1.54 ± 0.12^∗^	2.68 ± 0.49	2.90 ± 0.43	595.20 ± 40.16	5.82 ± 2.41^∗^	8026.93 ± 858.55^#^
CG	5.79 ± 0.23	466.17 ± 50.97	1.71 ± 0.13	2.58 ± 0.10	2.59 ± 0.37	604.26 ± 44.48^#^	30.5 ± 3.89^#^	8305.11 ± 482.49

The data was provided as mean ± standard deviation; ^∗^*p* < 0.05 versus the CG group; ^#^*p* < 0.05 versus the DM group.

## Data Availability

The data used to support the findings of this study are available from the corresponding author upon request.

## Additional Points

*Highlights*. This experiment explored the effect of vitamin D_3_ and IGF-1 on the testis of diabetic rats and the ways to regulate its biological effects, in order to improve the clinical diagnosis and treatment of diabetes.
